# Injuries of the Head

**Published:** 1829-07-01

**Authors:** 


### WESTMINSTER HOSPITAL.

### INJURIES OF THE HEAD.

Susan Randall, aged 4, admitted, having a wound of the head. She was playing with her brother, when he struck her on the head with a rake, one of the teeth of which pierced the cranium near the anterior fontenelle and opened the longitudinal sinus; she lost a good deal of blood and complained very much of pain in the head on first coming in, but shortly afterwards appeared rather lethargic.

Cold lotion to be applied to the head. Pulv. Rhei cum. Cal.

Feb. 1st.—There is still a good deal of pain in the head, particularly at the wound and in the forehead: there also seems to be a slight paralytic affection of the right arm and leg; she can move them, however, though not very freely. The powder to be repeated, and three leeches applied to the head.

4th. The pulsation of the arteries may be distinctly seen through the wound; pain in the head appears diminished except at the injured part. The leeches to be repeated.

5th. She is very lively to day; moves her right arm and leg more freely and seems free from pain.

9th. Does not complain of pain; can move her arms and legs with ease; there is a slight discharge from the wound.

11th. Complains of a little pain in her arm and head, especially when the wound is touched.—Pulv. Rhei cum Cal.

12th. Child seems a little better; a scab has formed over the wound.

14th. Matter collecting under the scab, which is therefore removed.

21st. Wound looks better; but the child is feverish.—Pulv. Rhei cum Cal.

23d. Wound has partly healed; a small sinus about half an inch in length has formed under the scalp which has this day been opened; the child looks very pale; bowels confined. — Haust. Cathart.

March 7th. Complains occasionally of a little pain in the head; the wound is healing up and discharges very little.

14th. The right leg swells a good deal, especially at night; she seems better.

26th. Both legs œdematous; the right heel is very much swelled and inflamed.

April 2d. Child is rather worse; looks very pale; does not sleep well at night; has no appetite; the right leg swelled, and an abscess is forming in the heel, which is to be poulticed. Is taking Pulv. Rhei cum Cal. et Potass. Supertart. daily.

4th. An abscess having formed in the heel it was opened and a good deal of matter discharged. To continue the poultice.

6th. Child is a little better, but still looks very ill; matter is again collecting under a scab which has formed over the wound; it is to be removed.

8th. Yesterday complained she could not see, and was continually rubbing her eyes; her bowels well open; she had a small blister applied to the nape of the neck, which has very much relieved the symptom; the abscess in the heel was re-opened as matter had again collected there.

From this time the child began gradually to recover; the swelling of the leg disappeared; the wound healed; her appetite improved; and in the beginning of May she was discharged quite well but looking rather pale.

Mr. Guthrie, in his clinical remarks on this case, drew the attention of the students to the fact that the longitudinal sinus had been opened, without much inconvenience ensuing. He said he had seen it happen several times, but without ever giving rise to serious annoyance,—the hæmorrhage stopping of itself in a short time. From the firm attachment of the dura mater to the bone, it did not allow of the formation of a coagulum sufficiently large to give rise to symptoms of compression. The partial commencing paralysis he rather attributed to irritation arising from the action of a slight irregular edge of bone on its internal surface. The surgery in this case he said consisted in examining the wound with a probe, in such a manner as to ascertain that no internal irregularity exists, in which case the common antiphlogistic means would alone be sufficient to effect the cure. When an irregularity or depression of the internal table is discovered, the bone should be immediately removed, so as to leave the dura mater, or the brain itself free from an irritation of that nature. The motions of the brain could in this case be seen very distinctly for several weeks, constituting, Mr. Guthrie said, altogether, one of the most valuable cases a student could see in the surgery of injuries of the head, more particularly when contrasted with others, then before them.

*Case* 2. Frank Collins, aged 30, admitted March 30th, having received three wounds on the head, made by the edge of a pewter pot. One on the left side of the frontal bone, divided the pericranium, and rendered the surface of the bone rough. The edges of the wounds were brought together by adhesive plaster, 30 ounces of blood were taken from the arm, and he was ordered to be severely purged, and to be starved.

On the 5th of April the wounds had united, and the patient left the hospital. 12th. Returned to shew himself quite well.

*Case* 3. John Esset, æt. 25, admitted March 27th, at 4, a. m. having fallen, when in a state of intoxication, from a window two stories high;—on examination, the left side of the face was very much bruised and there was a long, deep cut just below the eyebrow; the radius and ulna of the left arm were broken about two inches from the wrist. He had six leeches applied to his face. At 7 o'clock he fell asleep and awoke about 11, complaining of great pain in the left side just below the ribs, and headache; his pulse since his admission has been slow and compressible; he cannot lie in any position except on his right side.—

Venæ Sectio ad ℥xvj.

28th. Pulse much quicker, 80; pain in his head and side continues, but is not so severe; the whole face is much swelled; the wound to be poulticed.

Venæ Sectio ad ℥xvj.

℞ Liq. Amm. Acet. ℥ij.

Syrupi. ʒiv.

Aquæ ℥vj.

M. Cap. Cochl. iii. L. 4tâ. q.q. horâ.

30th. Pain in the head nearly gone; no pain in the side; bowels rather confined.—Pergat.

April 2d. Much better; the face and eyelids so much swelled, that the eye cannot be seen; wound is not looking very well; pulse sharp and quick. Pergat.

4th. The wound looking better; swelling diminished; no pain in the head; bowels open.

6th. The edges of the wound to be kept together by strapping. The arm has remained quite easy in the splints.

13th. Wound nearly healed; can just raise the eyelid, but finds that he cannot see with that eye; there is only a little speck on the cornea, and slight redness of the conjunctiva. He went out of the hospital at his own request a few days afterwards perfectly well in all respects, save that he had lost the sight of the left eye, without the slightest appearance of defect or disorganization about it.

Mr. Guthrie said that this case confirmed the observations he had made on the injury of the fifth pair of nerves, in his work on the Operative Surgery of the Eye. When treating of wounds of the eyelids he there stated, in opposition to all the opinions extant on the subject, that the pupil, in cases where blindness occurred from an injury of this nature, was not dilated, neither was it deficient in any of its sympathies, although it had been previously maintained that the pupil was dilated, and the iris deficient in its functions. This case, he said, proved the reverse, and therefore it was he begged their attention to this particular case.

*Case 4th.* William Walters, aged 10, admitted May 31st, under the care of Sir A. Carlisle. He fell 36 feet, from the roof of a house, upon his head. At the time of his admission he was rather in a comatose state; complained of pain in the head, and answered questions rationally. There is a considerable tumefaction in the occipital region on the right side of the head, and fluctuation is here very distinct; pulse low and rather irregular; about an hour and a half after his admission, however, his pulse rose to 120; cannot bear the light; vomiting came on.

Venæ sectio ad ℥vj.

Pulv. jalapæ comp. ʒss.

June 1st. Does not complain of any pain; bowels confined. Ordered an injection and purgative medicine.

2d. The tumour was opened and a quantity of bloody fluid discharged; the injection was repeated yesterday evening, but his bowels have not acted yet; the injections to be continued. Haust. olei ricini.

3d. Yesterday, at 4 o'clock, had a stool for the first time since his admission.

5th. Quite well; left the hospital.

